# Supplementary material for: Establishment of a Challenge Model for Sheeppox Virus Infection
Source: Microorganisms. 2020 Dec 15;8(12):2001. doi: 10.3390/microorganisms8122001 (PMC7765277; doi:10.3390/microorganisms8122001)
Supplement: Supplementary file 1 [file microorganisms-08-02001-s001.pdf]

Supplemental Table S1. Pan Capripox real-time qPCR results of different sample matrices after inoculation with SPPV-"India/2013/Surankote" via different inoculation routes. The genome copy numbers per µl template are presented. For calculation of the genome copy number per ml sample material, the listed genome copies must be multiplied by a factor of 400.

**SPPV-"India/2013/Surankote"**		**Genome copies/µl template at different days post inoculation**									
**Animal**	**Matrix**	**0dpi**	**3dpi**	**5dpi**	**7dpi**	**10dpi**	**12dpi**	**14dpi**	**17dpi**	**21dpi**	**27*/28dpi**
IND-IV-09	EDTA blood	no Cq	4,8E+00	1,3E+01	3,2E+01	2,0E+02	2,2E+02	7,3E+00	-	-	-
	serum	no Cq	4,3E-01	no Cq	no Cq	1,2E+00	3,6E+00	2,6E+00	-	-	-
	nasal swab	no Cq	no Cq	no Cq	1,6E+03	1,0E+03	3,1E+03	2,0E+04	-	-	-
	oral swab	no Cq	no Cq	no Cq	7,0E+00	1,1E+01	3,0E+01	1,1E+02	-	-	-
IND-IV-10	EDTA blood	no Cq	1,1E+00	1,9E+00	8,8E+01	7,4E+01	-	-	-	-	-
	serum	no Cq	no Cq	no Cq	no Cq	8,3E-01	-	-	-	-	-
	nasal swab	no Cq	no Cq	1,8E+00	8,2E+01	2,5E+05	-	-	-	-	-
	oral swab	no Cq	no Cq	no Cq	1,8E+00	2,3E+01	-	-	-	-	-
IND-IV-11	EDTA blood	no Cq	no Cq	2,7E+00	-	-	-	-	-	-	-
	serum	no Cq	no Cq	no Cq	-	-	-	-	-	-	-
	nasal swab	no Cq	no Cq	1,4E+00	1,2E+03	-	-	-	-	-	-
	oral swab	no Cq	no Cq	5,5E+00	no Cq	-	-	-	-	-	-
IND-IN-12	EDTA blood	no Cq	no Cq	8,1E-01	1,6E+01	4,7E+01	1,2E+01	9,5E+00	-	-	-
	serum	no Cq	no Cq	no Cq	no Cq	2,5E+00	no Cq	5,4E-01	-	-	-
	nasal swab	no Cq	6,3E+01	3,9E+05	4,3E+04	4,8E+05	4,8E+05	7,7E+04	-	-	-
	oral swab	no Cq	no Cq	1,6E+01	1,2E+02	5,3E+02	6,0E+03	2,7E+02	-	-	-
IND-IN-13	EDTA blood	no Cq	no Cq	1,3E+01	3,1E+01	6,4E+02	-	-	-	-	-
	serum	no Cq	no Cq	6,9E-01	no Cq	1,7E+01	-	-	-	-	-
	nasal swab	no Cq	9,8E+02	3,6E+03	1,1E+05	7,5E+06	-	-	-	-	-
	oral swab	no Cq	no Cq	9,3E+00	4,6E+02	1,0E+03	-	-	-	-	-
IND-IN-14	EDTA blood	no Cq	no Cq	no Cq	9,6E-01	5,8E-01	1,8E+00	no Cq	no Cq	no Cq	no Cq
	serum	no Cq	no Cq	no Cq	no Cq	no Cq	no Cq	no Cq	no Cq	no Cq	no Cq
	nasal swab	no Cq	1,0E+03	2,1E+02	8,2E+04	9,4E+05	5,2E+04	3,3E+03	1,5E+05	7,7E+04	1,2E+05
	oral swab	no Cq	no Cq	3,8E+00	7,7E+01	2,9E+01	3,2E+02	2,8E+02	6,3E+01	3,0E+02	6,5E+01
IND-IC-15*	EDTA blood	no Cq	no Cq	no Cq	no Cq	no Cq	5,6E-01	no Cq	no Cq	1,1E+02	7,0E+01
	serum	no Cq	no Cq	no Cq	no Cq	no Cq	no Cq	no Cq	no Cq	1,0E+00	3,5E+00
	nasal swab	no Cq	no Cq	no Cq	no Cq	4,3E+00	4,2E+01	5,4E+00	2,6E+01	6,3E+03	7,4E+03
	oral swab	no Cq	no Cq	no Cq	no Cq	2,4E+00	3,3E+00	3,1E+00	1,4E+00	1,3E+00	7,0E+03
IND-IC-16	EDTA blood	no Cq	no Cq	no Cq	no Cq	no Cq	no Cq	no Cq	no Cq	no Cq	no Cq
	serum	no Cq	no Cq	no Cq	no Cq	no Cq	no Cq	no Cq	no Cq	no Cq	no Cq
	nasal swab	no Cq	no Cq	6,1E-01	5,0E+00	2,4E+00	9,5E+01	5,0E+01	2,9E+00	1,1E+00	no Cq
	oral swab	no Cq	no Cq	no Cq	no Cq	no Cq	7,4E+00	1,5E+00	2,3E+00	1,1E+01	no Cq

Supplemental Table S2. Pan Capripox real-time qPCR results of different sample matrices after inoculation with SPPV-“Egypt/2018” via different inoculation routes. The genome copy numbers per µl template are presented. For calculation of the genome copy number per ml sample material, the listed genome copies must be multiplied by a factor of 400.

**SPPV-“Egypt/2018“**		**Genome copies/µl template at different days post inoculation**									
**Animal**	**Matrix**	**0dpi**	**3dpi**	**5dpi**	**7dpi**	**10dpi**	**12dpi**	**14dpi**	**16*/ 17dpi**	**21dpi**	**27*/ 28dpi**
EG-IV-01	EDTA blood	no Cq	no Cq	7,8E-01	5,6E-01	no Cq	1,4E+00	8,7E-01	no Cq	6,9E-01	no Cq
	serum	no Cq	no Cq	no Cq	no Cq	2,1E+00	7,7E-01	2,7E+00	no Cq	no Cq	no Cq
	nasal swab	no Cq	no Cq	no Cq	5,3E+00	1,3E+05	1,5E+05	3,3E+04	4,8E+02	1,0E+03	1,6E+02
	oral swab	no Cq	no Cq	no Cq	no Cq	1,1E+01	9,6E+01	2,3E+02	1,1E+02	4,0E+01	2,0E+00
EG-IV-02	EDTA blood	no Cq	no Cq	2,4E+01	7,1E+01	1,3E+02	1,2E+02	4,7E+02	-	-	-
	serum	no Cq	no Cq	1,5E+00	8,7E-01	5,1E+00	3,8E+00	1,6E+02	-	-	-
	nasal swab	no Cq	1,4E+01	4,1E+01	6,8E+01	6,2E+05	1,1E+05	1,5E+05	-	-	-
	oral swab	no Cq	no Cq	no Cq	1,3E+01	1,5E+02	1,9E+03	4,3E+02	-	-	-
EG-IV-03	EDTA blood	no Cq	2,8E+01	5,3E+00	5,2E+00	1,7E+00	no Cq	2,7E+00	no Cq	no Cq	no Cq
	serum	no Cq	3,2E+01	no Cq	no Cq	6,3E-01	1,2E+00	no Cq	no Cq	no Cq	no Cq
	nasal swab	no Cq	no Cq	no Cq	2,2E+01	5,7E+04	2,5E+04	2,0E+03	1,3E+02	1,0E+02	1,2E+02
	oral swab	no Cq	no Cq	no Cq	4,9E+00	1,2E+01	1,2E+02	3,8E+02	8,9E+01	1,6E+01	1,0E+00
EG-IN-04*	EDTA blood	no Cq	no Cq	7,9E-01	3,4E+00	1,1E+01	1,3E+00	no Cq	6,7E-01	-	-
	serum	no Cq	no Cq	no Cq	no Cq	5,1E-01	1,3E+00	no Cq	no Cq	-	-
	nasal swab	no Cq	6,1E+02	1,2E+02	5,6E+03	5,8E+05	2,1E+05	7,7E+05	6,0E+04	-	-
	oral swab	no Cq	no Cq	2,7E+00	2,2E+02	1,0E+03	1,8E+03	6,1E+02	5,7E+01	-	-
EG-IN-05*	EDTA blood	no Cq	no Cq	no Cq	no Cq	2,3E+00	no Cq	no Cq	2,2E-01	-	-
	serum	no Cq	no Cq	no Cq	no Cq	4,6E-01	1,1E+00	no Cq	no Cq	-	-
	nasal swab	no Cq	2,0E+02	2,3E+04	2,2E+04	3,1E+05	1,1E+06	2,7E+05	8,0E+05	-	-
	oral swab	no Cq	no Cq	1,9E+00	1,5E+01	1,1E+02	4,7E+02	2,8E+02	1,6E+02	-	-
EG-IN-06	EDTA blood	no Cq	no Cq	8,8E+00	7,3E+00	1,0E+01	1,8E+00	1,8E+00	-	-	-
	serum	no Cq	no Cq	no Cq	no Cq	1,8E+00	1,7E+00	4,6E-01	-	-	-
	nasal swab	no Cq	5,1E+00	5,8E+04	2,9E+05	1,0E+06	7,4E+05	2,2E+05	-	-	-
	oral swab	no Cq	no Cq	1,7E+01	1,4E+02	3,2E+02	3,4E+03	9,5E+02	-	-	-
EG-IC-07*	EDTA blood	no Cq	no Cq	no Cq	no Cq	no Cq	no Cq	no Cq	1,5E+00	3,5E+02	2,4E+01
	serum	no Cq	no Cq	no Cq	no Cq	no Cq	no Cq	no Cq	no Cq	4,4E+00	3,9E+00
	nasal swab	no Cq	no Cq	no Cq	1,3E+00	5,2E+00	3,9E+01	1,1E+01	6,3E+01	3,1E+05	8,9E+04
	oral swab	no Cq	no Cq	no Cq	no Cq	7,2E-01	1,1E+00	4,0E+00	2,0E+01	4,3E+01	6,4E+03
EG-IC-08	EDTA blood	no Cq	no Cq	no Cq	no Cq	8,4E+01	5,1E+01	3,2E+02	-	-	-
	serum	no Cq	no Cq	no Cq	no Cq	2,4E+00	no Cq	5,0E+00	-	-	-
	nasal swab	no Cq	no Cq	no Cq	no Cq	4,8E+01	1,4E+02	8,3E+05	-	-	-
	oral swab	no Cq	no Cq	no Cq	no Cq	no Cq	3,8E+00	2,5E+02	-	-	-

Supplemental Table S3. Pan Capripox real-time qPCR results of different tissue samples of sheep after inoculation with SPPV-"India/2013/Surankote" via different inoculation routes. The genome copy numbers per µl template are presented. For calculation of the genome copy number per g tissue material, the listed genome copies must be multiplied by a factor of 4000.

**SPPV-"India/2013/Surankote"**	**Genome copies/µl template**							
	**Intravenously**			**Intranasally**			**In-Contact**	
**Organ Sample**	**IND-IV-09**	**IND-IV-10**	**IND-IV-11**	**IND-IN-12**	**IND-IN-13**	**IND-IN-14**	**IND-IC-15**	**IND-IC-16**
cervical lymph node	5,0E+01	1,3E+01	2,0E+01	4,0E+00	1,5E+03	no Cq	7,6E+01	no Cq
mediastinal lymph node	no Cq	1,3E+01	5,0E+01	no Cq	no Cq	no Cq	no Cq	no Cq
mesenterial lymph node	no Cq	no Cq	no Cq	no Cq	no Cq	no Cq	2,6E+00	no Cq
liver	no Cq	no Cq	6,8E+00	no Cq	1,4E+00	no Cq	2,1E+00	no Cq
spleen	no Cq	no Cq	1,4E+00	no Cq	5,6E+00	no Cq	no Cq	no Cq
lung	5,2E+00	8,3E+02	1,2E+04	no Cq	1,2E+01	no Cq	9,5E-01	no Cq
**Additional Samples**								
coagulated heart blood			1,4E+02					
lung fluid			3,7E+01					
**Location of Skin Sample**								
breast		1,4E+02			2,6E+02			
		1,2E+05			1,4E+02			
		2,1E+02			1,0E+04			
		6,2E+04			3,0E+02			
axilla	8,5E+05	2,9E+02	3,7E+04	2,1E+00	5,5E+02		1,5E+03	
	1,2E+02	2,8E+03	4,0E+02	1,2E+00	6,1E+03		8,9E+03	
	5,9E+02	6,3E+01	7,3E+01	1,4E+00	5,0E+03		3,8E+03	
	1,6E+02	4,4E+04	5,4E+03	7,8E-01	8,1E+03		3,5E+02	
back					8,8E+03			
					1,3E+03			
					4,8E+05			
					6,6E+05			
hind leg		1,5E+01			3,5E+04		4,1E+04	
		3,0E+01			2,3E+05		1,0E+05	
		7,2E+01			2,5E+05		5,8E+04	
		2,5E+01			3,0E+05		1,3E+05	
tail							1,2E+05	
scrotum		7,9E+04			1,6E+03		4,1E+04	
					4,3E+02		1,0E+05	
prepuce		2,4E+05			4,1E+05		1,7E+05	
		2,7E+05					8,0E+05	
pox-like lesion lung	2,1E+00						2,2E+04	
	2,5E+00						7,5E+00	

Supplemental Table S4. Pan Capripox real-time qPCR results of different tissue samples of sheep after inoculation with SPPV-"Egypt/2018" via different inoculation routes. The genome copy numbers per µl template are presented. For calculation of the genome copy number per g tissue material, the listed genome copies must be multiplied by a factor of 4000.

**SPPV-"Egypt/2018"**	**Genome copies/µl template**							
**Organ Sample**	**Intravenously**			**Intranasally**			**In-Contact**	
	**EG-IV-01**	**EG-IV-02**	**EG-IV-03**	**EG-IN-04**	**EG-IN-05**	**EG-IN-06**	**EG-IC-07**	**EG-IC-08**
cervical lymph node	no Cq	2,54E+01	no Cq	2,30E+01	no Cq	no Cq	7,25E+02	2,19E+02
mediastinal lymph node	1,42E+00	no Cq	no Cq	7,71E-01	no Cq	no Cq	1,10E+01	3,31E-01
mesenterial lymph node	no Cq	1,05E+00	no Cq	no Cq	2,26E+00	no Cq	6,76E-01	no Cq
liver	no Cq	no Cq	no Cq	no Cq	no Cq	no Cq	no Cq	no Cq
spleen	6,18E-01	2,63E+01	6,60E-01	1,07E+00	no Cq	4,20E-01	2,37E-01	1,03E+00
lung	no Cq	7,36E+01	7,14E-01	7,57E+00	1,10E+01	5,50E+00	2,92E+00	8,89E+00
**Additional Samples**								
brain								no Cq
								no Cq
								no Cq
nasal septum				3,55E+04	3,51E+02			
**Location of Skin Sample**								
ear					7,94E+05			
					3,02E+06			
					1,18E+06			
foreleg							3,13E+05	
							7,05E+04	
axilla		1,90E+04						6,32E+03
		4,15E+04						1,66E+04
		2,11E+04						2,70E+02
		1,84E+05						
hind leg							9,98E+04	
tail							3,15E+05	
							1,65E+05	
							7,73E+04	
prepuce		1,03E+06		1,73E+04			6,05E+04	2,79E+05
		2,98E+06		8,67E+04			9,42E+05	
pox-like lesion lung				1,06E+03	1,44E+01			
				1,26E+03				
				4,75E+02				
healed skin lesion	3,47E+02		8,02E+03		5,30E+03			
	4,22E+02		9,70E+04					
	2,45E+01		3,62E+03					
	2,81E+02		7,70E+01					
	1,72E+03							
scab of skin lesion			5,47E+06					
			1,67E+06					
			1,27E+06					
			3,70E+05					
			4,48E+06					
